# Access to Drinking-water and Arsenicosis in Bangladesh

**Published:** 2006-09

**Authors:** Bruce K. Caldwell, Wayne T. Smith, Kamalini Lokuge, Geetha Ranmuthugala, Keith Dear, Abul H. Milton, Malcolm R. Sim, Jack C. Ng, S.N. Mitra

**Affiliations:** ^1^ National Centre for Epidemiology and Population Health, Australian National University, Canberra, Australia; ^2^ Centre for Clinical Epidemiology and Biostatistics, University of Newcastle, Newcastle, NSW, Australia; ^3^ School of Rural Health, Monash University, Bendigo, Australia; ^4^ Department of Epidemiology and Preventive Medicine, Monash University, Melbourne, Australia; ^5^ National Research Centre for Environmental Toxicology, University of Queensland, Brisbane, Australia; ^6^ Mitra and Associates, Dhaka, Bangladesh

**Keywords:** Arsenic, Arsenicosis, Drinking-water, Safe water, Tubewells, Water pollution, Water supply, Diarrhoea, Water testing, Bangladesh

## Abstract

The discovery of arsenic contamination in groundwater has challenged efforts to provide safe drinking-water to households in rural Bangladesh. Two nationally-representative surveys in 2000 and 2002 investigated water-usage patterns, water-testing, knowledge of arsenic poisoning, and behavioural responses to arsenic contamination. Knowledge of arsenicosis rose between the two surveys among women from 42% to 64% but awareness of consequences of arsenic remained limited; only 13% knew that it could lead to death. Behavioural responses to arsenic have been limited, probably in part because of the lack of concern but also because households are uncertain of how best to respond and have a strong preference for tubewell water even when wells are known to be contaminated. Further work conducted by the survey team highlighted the difficulties in providing alternative sources of water, with many households switching back to their original sources of water.

## INTRODUCTION

### Background: the need for safe water

The discovery that arsenic-contaminated groundwater is found in many tubewells in Bangladesh and in the neighbouring Indian state of West Bengal has provided a major challenge to the efforts to provide safe drinking-water. Tubewells in rural Bangladesh, which are safe from microbial contaminants, are the major source of water as these are much less expensive and easier to install than any other alternatives that might provide such water. To reduce the intake of arsenic will not be easy as alternative sources of water are generally more expensive, involve greater labour, can be less appealing to the village population, and, very importantly, often involve a greater risk of diarrhoea.

Before the adoption of tubewell water, diarrhoeal disease was a major cause of mortality, especially among young children. Although oral rehydration treatment (ORT), developed by the International Centre for Diarrhoeal Disease Research, Bangladesh (ICDDR,B), greatly reduced case mortality from diarrhoeal diseases, it did not reduce the incidence of diarrhoea. Furthermore, even with ORT, diarrhoea remains a major cause of mortality in Bangladesh.

Reduction of the incidence of diarrhoea requires the supply of safe water, sanitation, and good hygiene practices. While all three efforts are ideal, in Bangladesh, as in many other countries, the main focus is on the supply of safe water. The introduction of tubewells meant that safe water, at least in theory, was cheaper to provide than adequate sanitation and easier to address than hygiene, which requires extensive public education. Tubewells met this need for safe water because these were cheap (given Bangladesh's copious quantities of groundwater) and easy to install, required minimal maintenance, and provided microbially-pure groundwater directly to the household in plentiful quantities.

The spread of tubewells was indeed accompanied by a marked decline in mortality from diarrhoeal diseases, but much of this can be attributed to ORT and other treatments in reducing mortality. Early studies from the ICDDR,B field site at Matlab showed no difference in the incidence of diarrhoeal diseases due to water sources (1–3), or at most, showed a very limited protection in the form of a small benefit offered by drinking microbially safe water. Safe water for drinking was of minor significance because of the subsequent exposure to surface water through washing of utensils, preparation of foods, and washing of hands.

Results of subsequent international studies have suggested that supply of safe water significantly reduces the incidence of diarrhoeal diseases and, thus, mortality, but the absence of sanitation greatly reduces this positive effect. Esrey *et al.,* through a review of the available data, found that the provision of safe drinking-water and better sanitation reduced the incidence of diarrhoeal disease by 65% but that safe drinking-water by itself reduced mortality by only 20.8% (4). Emch found that, in Matlab, the use of tubewell water was associated with a significantly lower level of hospitalization for ‘non-cholera’ diarrhoea, but that sharing of sanitation facilities was a more important factor for hospitalization due to cholera (5). Access to improved sanitation is estimated at 41% in rural Bangladesh, implying that the remaining 59% have inadequate sanitation (6, 7).

### Impact of arsenic contamination

It is now clear that groundwater in the deltaic lands of Bangladesh and the neighbouring Indian state of West Bengal is contaminated by significant levels of arsenic (8–16). It has been estimated that 27% of shallow tubewells in Bangladesh, that is, wells not deeper than 150 metres, were contaminated with arsenic, exceeding the allowable Bangladesh maximum of 50 μg/L (50 parts per billion) and 46% of wells exceeded the World Health Organization's recommendation of 10 μg/L (10 parts per billion) (17). The equivalent figures for deep wells exceeding 150 metres were 1% and 5% respectively. It was estimated that 35 million people drank tubewell water that exceeded the Bangladesh maximum and 57 million drank water exceeding the WHO's recommended level.

A number of programmes have been launched to address the arsenic issue, most notably including the Bangladesh Arsenic Mitigation and Water Supply Project (BAMWSP), which is an initiative of the Government of Bangladesh with assistance from the World Bank. This project has most significantly conducted extensive testing of wells and encouraged the use of arsenic-free water sources. A number of non-government organizations (NGOs) are also developing locally-acceptable programmes to provide arsenic-free water (18).

### The issues

In this paper, we have examined water-usage by rural Bangladeshi people, their knowledge of arsenicosis and their response to this knowledge, and the health implications of adopting new sources of drinking-water. We drew on data from two national surveys of water-use conducted in 2000 and 2002, but reference was also made to a major intervention trial (19). The national surveys provide nationally-representative data on water-usage, plus an assessment of householders' understanding of arsenicosis and how they respond to it. The data should also allow an assessment of the burden of arsenicosis as a health issue in Bangladesh and the implications for the country's resources given competing health priorities.

## MATERIALS AND METHODS

We examined water-usage, knowledge of arsenicosis, early evidence of the effect of arsenic-reduction programmes, and some factors that inhibited their success. For this purpose, we used data from the Health and Social Research Project: Risks and Benefits of Arsenic Mitigation Programs in Bangladesh (HSRP). [The project was undertaken by a collaborative partnership of the National Centre for Epidemiology and Population Health, Australian National University; NGO Forum for Water Supply and Sanitation, Bangladesh; National Research Centre for Environmental Toxicology, University of Queensland; Department of Epidemiology and Preventive Medicine, Monash University; and Mitra and Associates, Dhaka.] The project had two key components, the first of which involved a nationally-representative sample survey of rural Bangladesh to examine the water-usage patterns and the response to the arsenic problem. The second key component involved an intervention study examining the risks and benefits of alternative interventions. The 2002 national survey was a follow-up of the 2000 national survey (20, 21). The 2000 survey involved interviews with respondents in 3,780 households that consisted of 20,260 individuals. Its sampling frame was a sub-sample of the 1996–1997 Bangladesh Demographic and Health Survey (22), which included 42 households drawn from each of 15 villages in each of the country's six divisions. In 394 cases (10.4%), the selected household was not available because there was no contact with any household member, there was no eligible respondent in the household, or the respondent refused. In these cases, subsitutions were made from a list of alternative households.

The 2002 survey sample consisted of a 30% sub-sample chosen randomly from the 2000 survey. In addition, from the earlier survey, all 46 households in which symptoms had been reported were included, resulting in a total of 1,181 households being identified for the 2002 survey. Of these, 61 households dropped out because they refused or because repeated visits failed to establish contact with any member of the household. Another 33 households were excluded because a person over 18 years of the identified sex (see below) was not available. This is equivalent to an overall response rate of 92.0% (n=1,087).

Fifty percent of interviews were conducted with male respondents and 50% with female respondents. The interviews were conducted, where possible, with the household head or spouse. By interviewing both men and women, it was possible to compare responses by sex. This distinction was important given the separate roles that men and women have in the household, notably in water management. In all interviews, female interviewers interviewed women and male interviewers interviewed men. The two surveys allowed an examination not only of the situation in rural Bangladesh as it was during the years in which the surveys were undertaken, but also the surveys allowed an examination of the changes in the intervening period, particularly in response to public-awareness campaigns and well-testing promoted by the BAMWSP (23). All data have been weighted by the population of the divisions to better represent the population distribution across the six divisions. In addition, a weighting has been used for taking into account the over-representation present in the second survey of households, which recorded skin rashes and lesions consistent with arsenicosis.

## RESULTS

The 2002 survey revealed that awareness of arsenic had increased since 2000 (Table 1). In 2000, 32.2% of the male and 22.3% of the female respondents were aware of arsenic; in 2002, 63.0% of the males and 59.9% of the females were aware of arsenic in water. The increase in awareness among women is particularly significant given that they are the principal users of water, and their cooperation is essential to any health strategy concerned with the appropriate use of water.

**Table 1.** Impact of information campaign (percentage of respondents)

Knowledge on arsenic problem	2000		2002	
	Male (n=1,890)	Female (n=1,890)	Male (n=541)	Female (n=543)
Has heard something may be wrong with tubewell water	47.5	41.8	68.6	63.9
Has heard of arsenicosis	32.2	22.3	62.9	59.8

Both male and female respondents with a formal education were much more likely to be aware of arsenicosis, although the educational differential had narrowed somewhat by 2002 (Table 2).

**Table 2.** Knowledge of arsenicosis by education (percentages)

Education (years)	2000		2002	
	Male	Female	Male	Female
0	18.4 (856)	12.3 (1,108)	52.5 (257)	48.0 (302)
1–5	27.8 (553)	26.2 (535)	61.6 (146)	67.9 (156)
6+	61.7 (481)	58.9 (246)	82.9 (140)	86.0 (86)
Total	32.2 (1,890)	22.3 (1,889)	62.9 (543)	59.7 (544)

*Figures in parentheses indicate denominators

The increase in awareness among women and less-educated respondents largely reflected an enhanced role of NGOs in raising awareness and an increase in community discussions with friends and neighbours (Table 3).

**Table 3.** Sources of arsenicosis knowledge (percentages of those who have heard of arsenic)*

Source of information	2000		2002	
	Male (n=609)	Female (n=421)	Male (n=340)	Female (n=325)
Government officer	9.2	5.5	2.6	4.6
Medical doctor	1.0	1.2	1.7	1.2
Government health worker	8.9	6.4	18.8	8.9
Non-governmental organization	14.0	19.5	11.5	32.3
Friend/neighbour	10.5	12.6	52.6	44.0
Relative	4.6	15.4	5.0	9.8
Family member	1.5	15.2	2.6	5.5
Radio	48.1	29.2	46.8	22.8
Television	50.1	44.4	58.8	44.3
Other	17.4	7.8	10.9	2.5

*Totals add up to more than 100 as more than one source is recorded

Most respondents did not know the health effects of arsenic, and only 12.9% were aware that arsenic could result in death (Table 4).

**Table 4.** Worst-perceived outcome of contaminated water (percentages)

Perception of arsenic-related health effects	2000		2002	
	Male (n=1,890)	Female (n=1,890)	Male (n=541)	Female (n=543)
Death	9.9	4.3	13.6	12.4
Permanent illness	4.7	4.7	10.4	5.7
Bouts of illness	15.3	13.1	19.9	13.2
Other/don't know	17.7	19.8	25.7	31.6
Total aware of arsenicosis	47.7	41.9	69.5	62.9

A key component in the Government's response to arsenic was testing wells. The surveys indicated that, while most wells remained untested, there had been a marked improvement from 5.7% tested in 2000 to 16.3% in 2002 (Table 5). This is well below the 50% reported for affected areas (24), but our survey included substantial areas outside the most affected areas. Nevertheless, even when taking this into account, there remained a worrying discrepancy.

**Table 5.** Wells tested for arsenic contamination by division (percentages)

Division	2000		2002	
	Male	Female	Male	Female
Dhaka	8.0 (527)	7.9 (529)	19.1 (152)	21.9 (151)
Chittagong	4.2 (336)	3.9 (336)	24.5 (98)	26.1 (111)
Rajshahi	0.6 (462)	0.4 (463)	1.4 (143)	4.3 (141)
Khulna	10.2 (216)	11.6 (216)	19.7 (61)	19.0 (63)
Barisal	12.2 (123)	13.8 (123)	18.8 (32)	26.3 (38)
Sylhet	2.0 (98)	3.2 (95)	14.3 (28)	12.9 (31)
Total	5.6 (1,762)	5.8 (1,726)	15.0 (514)	17.6 (535)

*Figures in parentheses indicate denominators

The 2002 survey identified that pumps used by 34.2% of the households interviewed were painted red, indicating that these exceeded the acceptable levels of arsenic, 27.3% were painted green to indicate these were acceptable, while 38.7% of the households had wells that had been left unpainted. It was unclear why these wells had not been painted.

Despite a great increase in awareness of arsenic, there had been only a moderate behavioural response towards the use of alternative water sources. In areas of high contamination of arsenic, the BAMWSP promoted the use of alternatives to tubewell water, such as sanitary dugwells, filtered pond-water, and deep tubewells, or, where these are not available, water filtered to remove arsenic. Deep tubewells are believed to be less dangerous than ordinary or shallow tubewells because they draw water from a deeper aquifer, which is usually much less contaminated by arsenic, although the required precise depth varies in the local aquifers (25, 26).

The data indicate that the use of tubewell water has not declined. In 2000, 87.2% of the respondents used shallow tubewells and 6.9% deep tubewells—the distinction between shallow and deep tubewells was self-defined and hence is only approximate. In 2002, 88.5% were using shallow tubewells and 7.4% deep tubewells (Table 6).

**Table 6.** Source of drinking-water (percentages)

Source	2000		2002	
	Male (n=1,890)	Female (n=1,890)	Male (n=541)	Female (n=543)
Tubewell	87.1	87.4	88.2	88.8
Deep tubewell	6.8	7.0	6.9	8.0
All other sources	6.1	5.6	4.9	3.2

The proportion of rural households not using either type of tubewell had declined from 5.9% to 4.1%. These findings are not surprising; these reflect a desire for households who can afford it to have their own source of water. Realistically, only inexpensive shallow tubewells provide such an option. Importantly, in contrast to the early 1970s when tubewells were promoted and paid for by the Government and international agencies, most wells are now privately owned by the household itself. Van Geen *et al.* have estimated that there are now up to 10 million tubewells, of which more than three-quarters are privately owned; in their own research area, 94% of tubewells were privately owned (25).

There is more to the desire to have one's own tubewell than simply the wish for a new prestige item in a country where few obvious consumer items can be afforded. Many continue to associate tubewell water with safe water, at least with regard to diarrhoeal disease, while women particularly want a water source that is convenient, given that collection of water can cut a great deal of their time, and one which they can control by having a tubewell installed in their own yard (20, 21).

Disputes over access to water are a source of village conflict. When households that had access to their own wells were asked in the 2002 survey what their main reason was for installing tubewells, there was a remarkable contrast between the answers of male and female respondents, reflecting different gender perspectives and experience on water (Table 7).

**Table 7.** Major reasons for installing a tubewell (households who have installed their own tubewells)

Resource	2000		2002	
	Male (n=876)	Female (n=840)	Male (n=265)	Female (n=280)
Safe drinking-water	61.2	42.7	70.3	31.1
Convenience	24.0	32.0	18.0	32.3
Can control one's own water supply	10.5	23.8	11.5	36.7
Other	4.3	1.6	0.3	0.0

Men were much more likely to give ‘safe drinking-water’ as their main reason, while women were equally likely to list ‘safe water’, ‘convenience’, and ‘the ability to control one's own water’. The men's answers appear to reflect past information campaigns, while women's answers reflect their greater role in water management and concern about practical issues that directly affect them. The implication of these responses is that if households need to be encouraged to switch away from using tubewell water, the message will need to be convincing, especially to women who are usually most concerned with water management.

An alternative to moving away from using tubewell water is for households to filter tubewell water to remove arsenic. This is potentially cheaper and more convenient than abandoning tubewells but also requires careful maintenance of the filter system, training, and at least some support in the initial stages. There is also a question regarding the long-term effectiveness of available filtering systems and the difficulties in using such systems for households (19, 27, 28). Perhaps for this reason, very few (0.6%) respondents filtered water for removing arsenic (Table 8).

**Table 8.** Overall any response to arsenic (percentages)

Option preferred	2000		2002	
	Male (n=1,890)	Female (n=1,890)	Male (n=541)	Female (n=553)
Changed water source	1.8	1.5	3.9	3.0
Filtered	0.2	0.3	0.8	0.4
Total	2.0	1.8	4.7	3.4

When asked why they did not treat their water, the majority of people in the 2002 survey stated that they did not know how to, while smaller proportions did not believe that their wells contained arsenic or they did not perceive arsenic to be a problem (Table 9).

**Table 9.** Main reasons for not treating water in 2002 (percentages)

Reason	Male (n=541)	Female (n=543)
Does not perceive arsenic to be a problem	10.7	5.5
Does not think that household tubewell contains arsenic	31.9	36.4
Does not know what to do	56.8	56.6
Other	0.6	1.4

The main behavioural response to arsenic identified was changing the water source. Nearly 90% of those who changed did so from one tubewell water source to another. This may be the most appropriate response. Considerable variability has been reported in levels of arsenic between neighbouring wells (17, 25). Provided that not too great a distance is involved, this solution offers lower arsenic intakes while retaining the advantages of tubewells in providing water that is free of microbial contamination. A number of researchers have recommended this approach (21, 29). Despite its attractions, the approach has not generally been favoured as there continues to be concerns about the reliability of testing and the fact that many wells falling below the Government arsenic standard contain some arsenic.

Switching from wells was the major response to arsenic recorded in the national survey. Households were asked whether they had previously used another tubewell. Nearly four-fifths (77.9%) had, but in only 11.1% of these households had those wells been tested. Where wells had been tested, households whose wells had been painted red were more likely to have shifted to other sources of water. Of the households whose previous wells had been tested, 57.3% had been painted red, 27.1% had not been painted, and 15.6% had been painted green. In comparison, the equivalent figures for current wells of households were: 34.5% wells were painted red, 27.5% wells painted green, and 37.4% wells not painted. Nevertheless, as these figures indicate, many households whose wells had been painted red continued to use them: 32.3% of all households whose current or previous wells had been tested for arsenic were currently drinking water from wells marked red. This means that 5.7% of the total population drank such water.

A prerequisite for a better response is the testing of more wells. While testing has increased, most wells remain untested. For people to respond adequately, they need to know whether their existing well is safe or not, and, if they prefer to use an alternative well, whether this will be safe. Although Cheng *et al.* have found only limited variability in concentration of arsenic over time (30), the process may also require re-testing of wells as it has been argued that levels of arsenic in wells may change.

However, the largest single factor preventing a behavioural response to arsenic is probably a lack of conviction that it is necessary or that it is in the interests of households to change their current water-use behaviour. While the respondents had heard of arsenic, the survey data did not suggest that they regarded it as an urgent health concern. Only 12.9% reported that arsenic could lead to death. Very few could identify people who were affected by arsenic, interests of households to change their current water-use behaviour. While the respondents had heard of arsenic, the survey data did not suggest that they regarded it as an urgent health concern. Only 12.9% reported that arsenic could lead to death. Very few could identify people who were affected by arsenic, and even fewer believed that a household member might be suffering from it.

The conditions to which arsenic poisoning may contribute, including internal cancers and cardiovascular diseases, also have other causes, and especially lay people may not believe that they are connected to arsenic. A long-latency period, in which arsenic-induced conditions may not manifest for decades, contributes to this confusion. It is partly for this reason that it took so long to realize the dangers of arsenic. The most obvious condition caused by arsenic is arsenicosis, which involves skin lesions that may, in due course, lead to skin cancer. There has been considerable debate about defining a diagnosis for arsenicosis and evaluating its link to more life-threatening conditions (31).

When asked whether anyone in their households had a condition caused by arsenic, only three respondents in 2002 said there was someone. Given the difficulty of defining arsenicosis, this number is not surprising, but it does emphasize the problem in convincing the average person that arsenic is a major health issue.

## DISCUSSION

The difficulties noted above in changing water-usage behaviour emphasize the need for clear and concise information on the most appropriate behaviour. This requires a better knowledge base than the one that currently exists. The suspicion expressed by many respondents that their health may suffer if they change their water source precipitously is not without foundation. Unless properly implemented, the dangers arising from arsenic mitigation may outweigh the benefits. The problem is that the risks are not all known and are difficult to quantify. As noted earlier, while the tubewell programme may not have been the major reason for the decline in mortality from diarrhoeal diseases, the evidence indicated that it has made a significant contribution (5). Given that diarrhoeal diseases remain a significant health danger, any programme that risks an increase in the incidence of diarrhoea would need to be significantly counter-balanced by an equal or greater reduction in arsenic-related morbidity and mortality in order to be justified (7).

### Issues concerning risks and benefits of arsenic intervention

Attempting to estimate the likely impact of diarrhoeal diseases is extremely difficult. In theory, a great deal should be known about the dangers of diarrhoeal diseases, but in practice, most studies on diarrhoeal diseases have examined incidence, while few have estimated mortality (7). Reliable estimates of incidence require large and representative sample sizes drawn from the community, as estimates based on hospital samples do not provide reliable community-level estimates. Data from the World Health Report suggest that 6.2% of all deaths in Bangladesh can be attributed to diarrhoeal diseases (32). Streatfield *et al.*, in an unpublished study, suggest that a much higher proportion of deaths (11.0%) can be attributed to diarrhoea (Streatfield K. Personal communication, 2006).

Arsenic-related morbidity is even more uncertain. While arsenic is well-known to be a deadly poison in high doses, there is less certainty about the degree of risk of human health effects at lower doses over a long time (7). Furthermore, unlike diarrhoeal diseases, which result in immediate acute illness lasting for one or two week(s) before recovery, the effects of arsenic can take decades to be manifested. Arsenic is carcinogenic and has been shown to cause several forms of cancer, including skin cancer and a number of internal cancers, such as lung and bladder cancers. It is calculated that arsenic will lead to a major increase in cancer-related deaths (33). It has also been related to high blood pressure and cardiovascular diseases. Nevertheless, because arsenic poisoning is only one factor contributing to both cardiovascular diseases and relevant cancers, it is difficult for epidemiologists to determine its precise contribution to the development of these health outcomes.

As part of the HSRP project, Lokuge *et al.* analyzed data from the scientific literature on what is known about arsenic as a cause of morbidity and mortality from cancer, cardiovascular diseases, and other health outcomes for given levels of arsenic intake (7) poorer health and death only in concentrations >50 μg/L. Based on this and what is known regarding the concentrations of arsenic in drinking-water in Bangladesh, they estimated that it contributed about 0.3% of the total burden of disease in Bangladesh. They concluded that, although it was a significant cause of burden of disease in the exposed population, it was less significant at the national level compared to many other risk factors. The lesson they drew was not that it could be ignored but that, since Bangladesh is a poor country with limited resources and multiple competing health problems, interventions need to be “targeted to those areas where exposure has been confirmed, and that those interventions provided achieve significant reductions in arsenic exposure without concomitantly causing substantial increases in other risks such as water-related infectious disease” (7).

Their calculations were complicated by the fact that much of morbidity and most of mortality caused by diarrhoeal diseases occur in young children aged less than five years, while the major effects of arsenic poisoning occur in older adults. Comparative measurements of health effects are most commonly calculated using disability-adjusted life-years, which strongly emphasize the impact of child mortality due to their greater number of years lost compared to what their disability-adjusted life-expectancy might otherwise have shown.

### Intervention study

While Lokuge *et al.* raise serious concerns about a response to arsenic contamination that is too broad and unfocused (7), these concerns also highlight the importance of ensuring that interventions are properly designed and have a sound scientific basis. Consequently, as one of its components, the HSRP undertook an intervention study of the health effects that the provision of two arsenic-mitigation interventions had on people compared to a control group. The two interventions included the provision of sanitary dugwells to provide an arsenic-free source of water and a ‘three-pitcher’ filter system to remove arsenic from tubewell water. [This filter system contains three clay-pots stacked on top of one another, with two top pots containing filtration media and precipitants to filter water. The primary active ingredient is iron filings to which arsenic binds. The resultant precipitate is filtered out]. The sanitary dugwells and the ‘three-pitcher’ filter system are two of the most widely-promoted interventions in Bangladesh and represent the two main approaches to arsenic mitigation.

The project installed dugwells or provided three-pitcher filter systems to households in the intervention areas and provided training to caretakers on maintaining the system. It also provided health education on management of diarrhoeal diseases to ensure that the interventions had no adverse consequences of mortality: the factor being measured was cases of diarrhoea, not mortality from diarrhoea. In an analysis of the findings, Milton *et al.* found significant difficulties in compliance in using either of the two methods, especially dugwells, with households falling off badly and reverting to tubewell water (19).

The discovery of arsenic in groundwater as a serious health issue in Bangladesh concerns the role of the Government, international agencies, and public-health experts, in promoting the use of groundwater through tubewells as a safe-water option. Reduction in mortality in developing and developed countries in recent decades has been possible due to increased knowledge of disease transmission, how to prevent disease, and how to treat it. An important part of this achievement has been the reduced transmission of waterborne diseases. Bangladesh has been a particularly impressive example given its initial disadvantage of having a large and resource-poor population. The discovery of arsenic-contaminated groundwater has been a major setback to its health programme, but the key point is to learn from an objective examination of the evidence.

Concern about safe water is only one factor that affects the way people behave with regard to water. It is important not just for health professionals but also for household members themselves. Household members, especially women, are concerned about family well-being and health. Despite their concern, there are many other obstacles to changing their water-use behaviour, including the desire, especially of women, to maintain control over water via tubewells and the easy access they have to tubewells. BRAC found that villagers were reluctant to accept methods that they felt were going back to old and discarded ways, but they were more willing to accept methods they regarded as forward-looking, such as piped water and deep tubewells (18).

However, most alternatives, such as piped water, are likely to be costly in terms of money, time, and effort. Even using alternative tubewells involves a cost if those wells are away from the household and are, therefore, inconvenient. Any future intervention will need to address these issues and especially be sensitive to gender differences in water usage and control. It will be especially important to ensure that any solution involving shifting away from tubewell water does not lead to increased incidence of diarrhoeal diseases.

Arsenic remains a substantial issue for Bangladesh. Millions of people drink water that exceeds the Government-recommended maximum arsenic level of 50 μg/L, and millions more drink water that exceeds the international maximum of 10 μg/L. It remains critical to test all currently-used wells in areas with potentially high levels of arsenic and, where feasible, potential alternative wells. For those households whose wells record high levels of arsenic, testing nearby wells may provide an alternative source of safe water. Since the costs of testing are much lower than the costs relating to household labour and financial matters and the convenience involved in using alternative water sources, this would appear to be the most viable strategy.

Where it is not possible for households to use water from their own tested tubewells or other tested tubewells, alternative options will be needed. However, it will not be simple to have alternative options accepted on a permanent basis. If people are to be encouraged to move to alternative sources of water, there are a number of critical issues. Any alternative for providing ‘adequate safe’ water will generally be much more expensive and will require maintenance and, in consequence, will generally be shared by several households. This raises the issues as to who will pay, and how payment, access, and responsibility for maintenance will be shared.

Crucially, any efforts to encourage householders to shift to alternative sources of water will need to be accompanied with programmes to reduce the incidence of diarrhoea. The new sources of water should be designed to be as safe from microbial contamination as possible, but it will also be important to encourage a better understanding of the importance of hygienic handling of water. This also applies, but to a lesser extent, to those who continue to use tubewell water.

## ACKNOWLEDGEMENTS

This research was funded by a grant from AusAID (Australian Agency for International Development) to NCEPH (National Centre for Epidemiology and Population Health) at the Australian National University. The authors thank Professor Bob Douglas and Professor Jack Caldwell for their contributions in undertaking the initial National Survey in 2000.
